# More Is Not Always Better: Co-Occurrence Analysis of Anti-Phage Systems Reveals Prevalent Neutral Interactions in *P. aeruginosa*

**DOI:** 10.3390/microorganisms13122783

**Published:** 2025-12-07

**Authors:** Shuhong Han, Xiaofu Wan, Zixun Lin, Xiangke Duan, Yukun Zeng, Jiayin Shen, Hongzhou Lu

**Affiliations:** 1 National Clinical Research Center for Infectious Diseases, The Third People’s Hospital of Shenzhen and The Second Affiliated Hospital of Southern University of Science and Technology, Shenzhen 518112, China; hansh1010@163.com (S.H.); wanxf15@126.com (X.W.); 12531548@mail.sustech.edu.cn (Z.L.); xkeduan@163.com (X.D.); zengyk1992@126.com (Y.Z.); 2 School of Medicine, Southern University of Science and Technology, Shenzhen 518055, China

**Keywords:** *Pseudomonas aeruginosa*, anti-phage, prokaryotic immunity, co-occurrence, neutral effects

## Abstract

*Pseudomonas aeruginosa* is a major nosocomial pathogen and a key model for studying phage–bacteria interactions. While a multitude of anti-phage defense systems have been discovered in this species, the functional relationships between them remain poorly characterized. Here, we sequenced 53 clinical *P. aeruginosa* isolates and uncovered an arsenal of diverse defense systems. Surprisingly, phage infection and adsorption assays revealed that the quantity of defense systems in a given strain did not correlate with its innate phage resistance. Subsequent bioinformatic analysis identified statistically significant positive and negative associations between specific defense systems. Experimental validation of selected pairs demonstrated that certain pairs conferred a spectrum of interaction outcomes, including additive, synergistic, and neutral effects, clearly demonstrating that the outcome cannot be predicted by a simple additive model. Our findings establish that functional relationships between defense systems, rather than their mere abundance, is a critical determinant of the anti-phage defense landscape in clinical *P. aeruginosa*, with implications for bacterial evolution and phage therapy.

## 1. Introduction

The global rise in antibiotic resistance has spurred a renewed interest in bacteriophage (phage) therapy, evaluating its potential to supplement or even replace conventional antibiotic [1,2,3,4]. The clinical translation of phage therapy, however, is challenged by the sophisticated and diverse arsenal of intracellular immune mechanisms that bacteria employ to counteract phage infection. While well-characterized systems, such as restriction-modification (RM) and CRISPR-Cas, illustrate fundamental principles of antiviral defense [5,6,7,8], they represent only a fraction of the known anti-phage arsenal.

Bacterial genomes often organize these defense mechanisms into genomic “defense islands” [8], where they exhibit non-random co-occurrence patterns across diverse bacterial taxa [9,10,11,12,13,14]. Furthermore, the component of defense systems is highly variable, even among conspecific strains; a typical bacterial genome encodes an average of five identifiable systems [15]. Despite this prevalence, the functional relationships and potential interplay between co-resident systems within natural bacterial genomes are poorly defined.

*Pseudomonas aeruginosa* (*P. aeruginosa*) is a Gram-negative bacterium with notable ecological plasticity and a facultative anaerobic nature [16,17], representing a formidable pathogen in the post-antibiotic era. *P. aeruginosa* pangenome is remarkably enriched with diverse and acquired defense systems, such as CBASS, RM, and Gabija [18]. This combination of acquired and intrinsic anti-phage systems makes *P. aeruginosa* an exceptional model for elucidating the complexity of bacterial immunity against phages. However, how these systems engage in functional interactions to ultimately shape bacterial fitness remains largely unexplored.

Herein, we conducted a genomic analysis of 53 clinical, multidrug-resistant *P. aeruginosa* isolates with a comprehensive infection panel of 40 phages spanning six phylogenetic groups. We discovered that the sheer number of defense systems in a genome was a poor predictor of innate phage resistance. Systematic co-occurrence analysis revealed statistically significant associations between specific defense systems. Experimental validation of selected pairs confirmed a spectrum of interaction outcomes, clearly demonstrating that the outcome cannot be predicted by a simple additive model. Our study provides direct experimental evidence for functional collaboration within anti-phage defense networks, underscoring the importance of understanding the system-level relationships that dictate the success of phage therapy against resilient clinical pathogens.

## 2. Materials and Methods

### 2.1. Bacterial Strains and Phages

A complete list of the bacterial strains, phages, plasmids, and all primers used in this work are listed in the following Supplementary Tables S1–S3, S5 and S6. Carbenicillin (150 μg/mL) was used for *P. aeruginosa* strains. Ampicillin (100 μg/mL) was used for *Escherichia coli* (*E. coli*) strains. Gene expression was induced by the addition of 0.4% *L*-arabinose when needed.

### 2.2. Bacterial Genomes Collection

A collection of 1102 complete *P. aeruginosa* genomes used in this study were obtained from the NCBI RefSeq database on 1 September 2025. The dataset was filtered to include only genomes designated as “Complete Genome” and “Reference” to ensure high quality. 53 clinical *P. aeruginosa* isolates were collected from our hospital.

### 2.3. Genome Sequencing and Bioinformatic Analysis

Genome sequencing and bioinformatic analysis were conducted as previously described [19]. Briefly, both bacteria and phage genome sequencing was performed by Illumina Hiseq 2000 sequencer (San Diego, CA, USA). Raw reads were quality-trimmed and de novo assembled using Shovill v1.0.4. Contigs shorter than 500 bp or with a coverage of <2× were discarded. The final assembly was assessed with Quast, and phage taxonomy was assigned using PhaBOX (https://phage.ee.cityu.edu.hk/, accessed on 26 August 2025) [20]. The raw sequencing data for the clinical isolates generated in this study have been deposited in the China National GeneBank Database (CNSA) with accession number CNP0008262.

### 2.4. Phylogenetic Analysis

The Average Nucleotide Identity (ANI) analysis based on whole genomes was performed by FastANI v1.3 to infer the phylogenetic relationships among the 53 clinical strains and PAO1 reference genome (RefSeq: NC_002516.2) [21]. The Neighbor-Joining (NJ) tree was constructed from the ANI distance matrix in MEGA7 [22]. The generated phylogenetic tree was then tested using the Bootstrap method with 1000 replications [23], and visualized in iTOL (https://itol.embl.de/, accessed on 19 October 2025).

### 2.5. Identification of Anti-Phage Immune Systems

**Figure 1:**
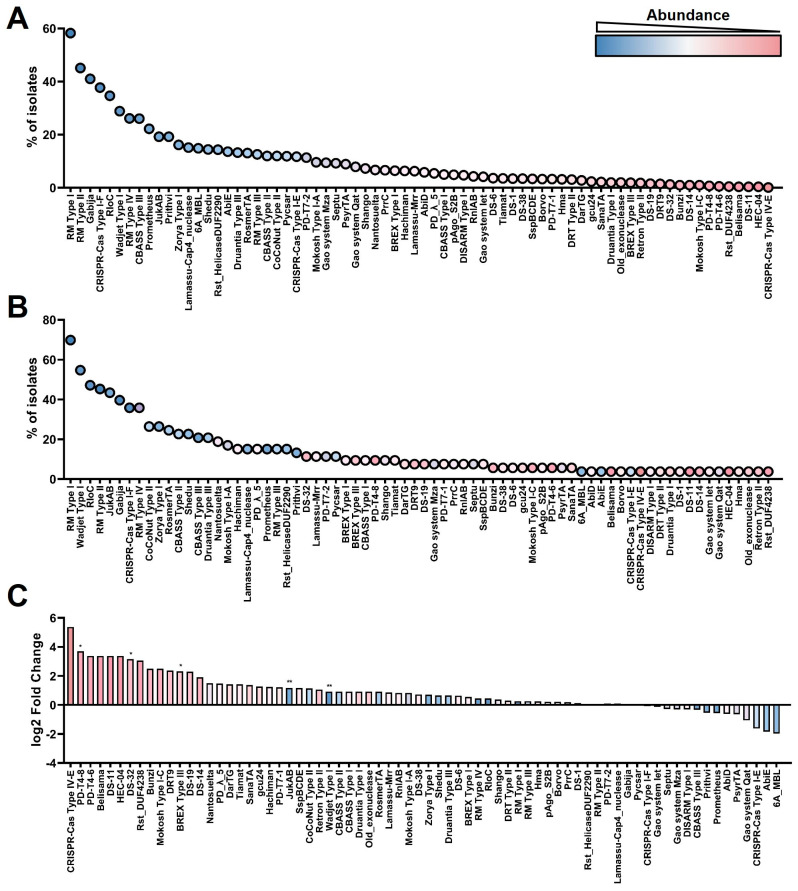
Comparative analysis of defense system abundance and diversity in *P. aeruginosa* genomes. (**A**) Prevalence of defense systems in 1102 *P. aeruginosa* genomes from the RefSeq database. Systems are ordered from most to least abundant (left to right) and colored on a gradient from blue (high abundance) to pink (low abundance). Only the most prevalent defense systems are shown (see Table S1 for the full list of defense systems). (**B**) Prevalence of defense systems in 53 clinical isolates from this study. Systems are ordered and colored identically to (**A**) to facilitate comparison (see Table S2 for the full list of defense systems). (**C**) Fold-change (FC) in the abundance of each defense system in clinical isolates relative to the RefSeq baseline. Significance was evaluated using Fisher’s exact test (* *p* < 0.05 and ** *p* < 0.01).

**Figure 2:**
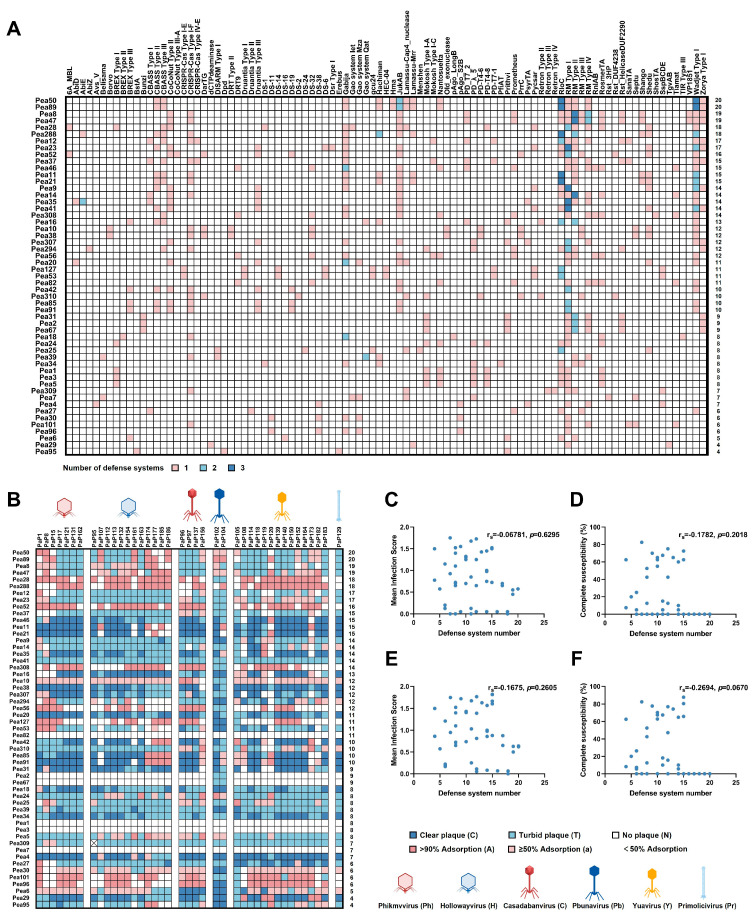
Analysis of defense system repertoire and phage susceptibility in clinical *P. aeruginosa* isolates. (**A**) Distribution of anti-phage defense systems across clinical isolates. Heatmap displaying the abundance of various anti-phage defense systems in 53 clinical isolates. Abundance levels are represented by a color gradient: dark blue (value = 3), light blue (value = 2), and light pink (value = 1). Rows correspond to individual bacterial strains, and columns represent distinct types of defense systems, sorted in descending order of prevalence. The total number of systems identified per strain is indicated on the right. Detailed strain information is provided in Table S2. (**B**) Host range of phages against 53 clinical *P. aeruginosa* isolates. The phylogenetic clustering of phages was detailed in Table S3. Phage–bacteria interactions are represented by a color spectrum: dark blue indicates the formation of clear plaques (C); light blue indicates turbid plaques (T); dark pink represents adsorption ≥ 90%; light pink represents adsorption ≥ 50%; and white indicates no plaque formation or adsorption < 50%. Rows correspond to individual bacterial strains, and columns represent distinct phages. (**C**) Correlation between the total number of defense systems per strain and its mean infection score (N = 0, T = 1, C = 2) across all phages for the entire strain collection (Spearman’s r = −0.06781, *p* = 0.6295). (**D**) Correlation between the total number of defense systems per strain and the number of phages producing clear plaques (C) for the entire strain collection (Spearman’s r = −0.1782, *p* = 0.2018). (**E**) Correlation between the total number of defense systems and the mean infection score, analyzed exclusively for the subset of strains exhibiting normal adsorption (≥50%) (Spearman’s r = −0.1675, *p* = 0.2605). (**F**) Correlation between the total number of defense systems and the number of phages producing clear plaques, analyzed exclusively for the subset of strains exhibiting normal adsorption (≥50%) (Spearman’s r = −0.2694, *p* = 0.0670).

A comprehensive analysis of anti-phage defense systems in *P. aeruginosa* strains was conducted using DefenseFinder with default parameters [15], and the identified systems are presented in Tables S1 and S2, along with Figure 1 and Figure 2A.

### 2.6. Phage Host Range

The host range of the phage was determined against each bacterial strain using a spot assay to evaluate lytic activity. Bacterial lawns were prepared by mixing exponential-phase cultures (OD_600_ ~0.8) with soft agar, the purified phages were spotted on the upper layers. Following incubation, plaque morphologies (characteristics and size) were systematically documented. This assay was independently repeated three times and only results consistent in at least two replicates were considered conclusive.

### 2.7. Adsorption Assays

Bacterial cultures in the exponential phase (OD_600_ ~0.8) were dispensed into a 96-well plate, followed by infection with phages using a multiplicity of infection (MOI) of 0.01. Following a 15 min adsorption period at 37 °C with shaking, the samples were centrifuged. The supernatant was diluted and spotted on the lawn of PAO1 to quantify the no-adsorbed phages. The percentage of adsorbed phages was calculated as [1 − (PFU in supernatant/PFU in initial inoculum)] × 100%. Phages were considered to have adsorbed effectively if the calculated percentage exceeded 50%. Experiments were performed in triplicate.

### 2.8. Analysis of the Co-Occurrence and Negative Association Among Defense Systems

To elucidate the interaction among the systems, a correlation analysis was conducted on the presence–absence matrix comprising 95 defense systems across 53 samples using XLSTAT software (version 27.1.3.0) (Addinsoft Corporation, https://www.xlstat.com/), accessed on 11 September 2025. Pairwise Pearson correlation coefficients were calculated, the statistical significance of these correlations was evaluated using both the stringent Bonferroni correction (for primary analysis) and the less strict Benjamini–Hochberg false discovery rate (FDR) (for exploratory analysis). Significant correlations were ranked by the absolute value of the correlation coefficient, with Bonferroni-corrected associations prioritized for higher reliability. Network of co-occurrence and negative association was visualized in Cytoscape v3.10.3 [24].

### 2.9. Cloning of Defense Systems in PAO1

The plasmids and their corresponding primers used in this work are provided in Table S5 and Table S6, respectively. The PCR products were amplified by Q5^®^ High-Fidelity 2X Master Mix (New England Biolabs, Ipswich, MA, USA) and ligated to the EcoRI- and HindIII-digested vector pHERD20T using Gibson Assembly master mix (New England Biolabs, Ipswich, MA, USA). PCR combined with Sanger sequencing was employed to confirm the successful construction of the recombination plasmids. Next, all recombination plasmids were transferred into bacterial hosts via electroporation (Bio-Rad Laboratories, Hercules, CA, USA).

### 2.10. Efficiency of Plating (EOP)

Bacterial cultures were grown to exponential phase (OD_600_ ~0.8) in LB containing 150 μg/mL carbenicillin and induced with 0.4% arabinose for 2 h. The induced cultures were then subjected to a spot assay to determine phage efficiency of plating (EOP). Epistatic interactions between defense systems were quantified as previously described [12]. Neutral effects was defined when the resistance level of the co-expressed pair was statistically indistinguishable from that of the stronger single system alone; synergy was defined when |Log_10_ (EOP_combined_)| > |Log_10_ (EOP_system1_)| + |Log_10_ (EOP_system2_)| + 1; additivity was defined when the combined effect was greater than the effect of the stronger single system but did not meet the threshold for synergy; marginal additivity was defined as a combined effect that conferred precisely a one order of magnitude enhancement over the stronger system; antagonism was defined when the combined system exhibited a resistance level more than one order of magnitude weaker than that of the more effective single system. This assay was independently repeated three times and only results consistent in at least two replicates were considered conclusive.

### 2.11. Quantitative Reverse Transcriptase PCR (qRT-PCR)

Gene expression in *P. aeruginosa* harboring different plasmids was analyzed by RT-qPCR. Bacterial cultures were diluted to OD_600_ 0.01 and grown to the exponential phase with or without 0.4% L-arabinose induction. Total RNA was extracted using RNeasy minikit (Qiagen, Hilden, Germany) and reverse-transcribed into cDNA. qPCR was performed in 20 µL reactions using SYBR Green Master Mix (Vazyme), Nanjing, Jiangsu, China on the LightCycler 96 system (Roche, Basel, Switzerland) under standard cycling conditions. All reactions were run in triplicate, and all primers are listed in Table S6.

### 2.12. Growth Assay

*P. aeruginosa* carrying different plasmids were inoculated in triplicate at an initial OD_600nm_ of 0.01 and incubated statically at 37 °C. The OD_600nm_ was measured every 15 min for 12 h using the microplate reader (Tecan, Männedorf, Switzerland), and the data were used to plot growth curves.

### 2.13. Time Post Infection Assay

*P. aeruginosa* carrying different plasmids were diluted to an OD_600nm_ of 0.01 and induced with 0.4% *L*-arabinose until OD_600nm_~1.0. Following infection with phage at an MOI of 0.01, the cultures were incubated at 37 °C with shaking at 150 rpm. A sample was taken at 0, 1, 2, 3, 4 and 5 h post infection to determine the absorbance at 600 nm.

### 2.14. Data Analysis and Statistics

Unless otherwise specified, data analysis was made using SPSS 20, and all figures were created using GraphPad Prism v8.0.2.

## 3. Results

### 3.1. Clinical P. aeruginosa Strains Exhibit Abundant and Diverse Defense Systems

To establish a comprehensive profile of defense system distribution in *P. aeruginosa*, we first analyzed a curated dataset comprising 1102 high-quality genomes from the RefSeq database (accessed on 1 September 2025). Our analysis identified 186 distinct defense systems across the analyzed genomes, representing 70.5% (186 of 264) of the known defense system subtypes, a proportion higher than any previously reported one [25]. And the most prevalent systems included RM Type I (58.26%), RM Type II (45.10%), Gabija (41.02%), and CRISPR-Cas Type I-F (37.75%) (Figure 1A, Table S1), a hierarchy consistent with earlier studies [25]. This conservation in the core architecture of defense systems supports the validity of our analytical methodology and underscores the stability of these systems across expanded genomic datasets.

We next sequenced 53 clinical *P. aeruginosa* isolates from our hospital (collected between January 2019 and September 2025) (Figure 1B, Table S2) and calculated the fold change (FC) in the abundance of each defense system relative to the updated RefSeq baseline (Figure 1C). This comparison revealed significant enrichment of several defense systems in the clinical isolates, including PD-T4-8 (9.43%), DS-32 (11.32%) and BREX Type III (9.43%).

To evaluate the strain-specific defense potential, we quantified the number of defense systems per genome. The updated RefSeq collection revealed a median of 9 defense systems per genome (Figure S1A,B), exceeding the previously reported median of 7 [25]. Notably, the clinical isolates harbored a median of 11 systems per genome (Figure S1A,C), suggesting that clinical strains may accumulate more defense systems under persistent selective pressures. Phylogenetic analysis based on whole-genome sequences classified the 53 clinical isolates into three distinct clades (Figure S1D). We observed that genomes in Clade II contained more defense systems than those in Clade III, which includes the reference strain PAO1 (Figure S1E).

Collectively, our integrated analysis of an updated global dataset and newly sequenced clinical isolates not only reaffirmed the core anti-phage defense architecture of *P. aeruginosa* but also revealed a broader diversity of systems and a general trend of defense enrichment in clinical strains.

### 3.2. The Breadth of Phage Resistance Is Not Solely Determined by Their Defense System Number

The considerable inter-strain variation in the number of defense systems, and their presence–absence profiles suggested the existence of distinct evolutionary lineages within the population (Figure 2A). To functionally assess these defense systems, we used a subset of 40 phages in our laboratory collection that were previously isolated from sewage water (Table S3). 39 phages belonged to the class *Caudoviricetes* [double-stranded DNA (dsDNA) tailed phages], including 4 *Casadabanvirus*, 12 *Hollowayvirus*, 2 *Pbunavirus*, 7 *Phikmvvirus*, and 14 *Yuavirus*. One additional isolate was classified as *Primolicivirus* within the class *Faserviricetes* [circular single-stranded DNA (ssDNA) phages]. We performed plaque-forming unit (PFU) assays to distinguish productive infections from mere phage adsorption. Across 2120 phage–host pairs (40 phages × 53 clinical strains), we observed a spectrum of infection outcomes, ranging from full susceptibility (clear plaques), partial resistance (turbid plaques), to complete resistance (no plaques). Infection failed in 959 of these interactions. The resulting phage–host infection matrix illustrated the broad variability in strain-specific susceptibility (Figure 2B).

To investigate the correlation between the number of defense systems per strain and its overall phage resistance, we determined a mean infection score for each strain. Outcomes were assigned ordinal values as follows: no plaque (N) = 0, turbid plaque (T) = 1, clear plaque (C) = 2. The mean score in each strain, which was determined across 40 different phages, reflected overall susceptibility, with lower values indicating stronger resistance. Interestingly, a lack of significant correlation between the total number of defense systems and the mean infection score was observed (Spearman’s r = −0.06781, *p* = 0.6295; Figure 2C). Nor correlation was detected when the fully susceptible interactions were only considered (i.e., clear plaques; Spearman’s r = –0.178, *p* = 0.202; Figure 2D).

Given that adsorption failure constitutes first line of resistance independent of intracellular immunity [11], we performed quantitative phage adsorption assays to exclude cases where infection failure stemmed from receptor recognition defects. Six strains were excluded from subsequent analysis (Pea1, Pea2, Pea3, Pea7, Pea67, and Pea82), as they showed near-complete adsorption defects. A revised correlation analysis on the remaining adsorption-competent strains still revealed no significant association between defense system count and either the mean infection score (Spearman’s r = –0.168, *p* = 0.261; Figure 2E) or the number of phages forming clear plaques (Spearman’s r = –0.269, *p* = 0.067; Figure 2F).

### 3.3. 107 Pairs of Defense Systems Co-Occurred in Clinical P. aeruginosa Strains

**Figure 3:**
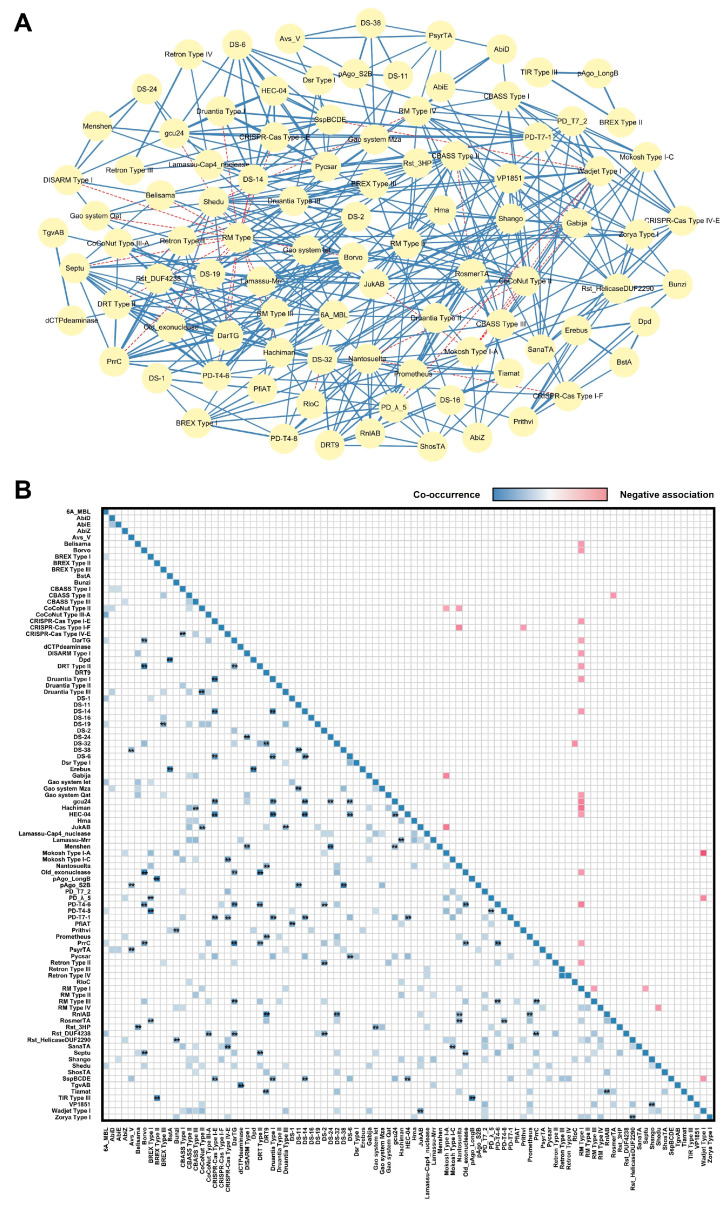
Co-occurrence and negative association between defense systems in clinical *P. aeruginosa* isolates. (**A**) Network of co-occurrence and negative association between defense systems. Nodes represent individual systems. Edges represent significant correlations after Benjamini–Hochberg FDR correction, co-occurrences are shown in blue solid line, while negative associations are shown in pink dash line. Edges thickness is proportional to the absolute value of the correlation coefficient. (**B**) Correlation analysis of defense system across clinical *P. aeruginosa* isolates. Hierarchically clustered heatmap of pairwise Pearson correlation coefficients for defense system pairs. The Pearson correlation coefficient, mathematically equivalent to the Phi coefficient for binary data, was used to quantify associations. Co-occurrences are shown in blue, while negative associations are shown in pink. Asterisks (**) indicate systems that remained significantly co-occurring after the most stringent Bonferroni correction. Comprehensive information is presented in Table S4.

Cooperative interaction between defense systems has been found in *E. coli* and *Escherichia fergusonii* (*E. fergusonii*), thereby enhancing or expanding protection against phages [12,15,26,27,28,29,30]. To investigate whether such qualitative interactions would be found in *P. aeruginosa*, we systematically analyzed the relationships (which could be positive, or negative or neutral) across all pairs of defense systems in our clinical *P. aeruginosa* collection. This approach was motivated by the premise that strong co-occurrence may indicate functional coupling or mechanistic interdependence that directly contributes to bacterial immunity [12]. We quantified associations using the Pearson correlation coefficient, a mathematically equivalent to the Phi coefficient for binary data, to assess the strength and direction of each pairwise relationship. 107 defense system pairs (1.2% of all pairs analyzed) exhibited a significant positive correlation after Bonferroni correction for multiple testing (Figure S2A, Table S4). Under a more lenient Benjamini–Hochberg FDR correction, 402 pairs (4.5%) showed strong correlation, of which 372 (372/402, 92.5%) were co-occurring (Figure 3A and Figure S2B, Table S4). The resulting association network displayed a clear modular architecture (Figure 3B), with several densely interconnected clusters of co-occurring systems.

The high positive correlation found in some pairwise combination, including but not limited to Druantia Type III- JukAB (r = 0.58, *p* = 4.32 × 10^−6^) and CBASS Type III-Hachiman (r = 0.56, *p* = 1.09 × 10^−5^), nicely exemplified a potential selective advantage for their co-inheritance. In contrast, Mokosh Type I-A and Wadjet Type I showed a significant negative correlation (r = −0.50, *p* = 1.52 × 10^−4^), indicative of possible genetic incompatibility or redundancy in functionality. Altogether, the network was overwhelmingly dominated by significantly co-occurring system pairs, with negative associations being comparatively rare. This may reflect the adaptive evolution of cooperative anti-phage strategies.

### 3.4. Co-Occurrence of Defense Systems Confers Diverse Outcomes in Anti-Phage Immunity

**Figure 4:**
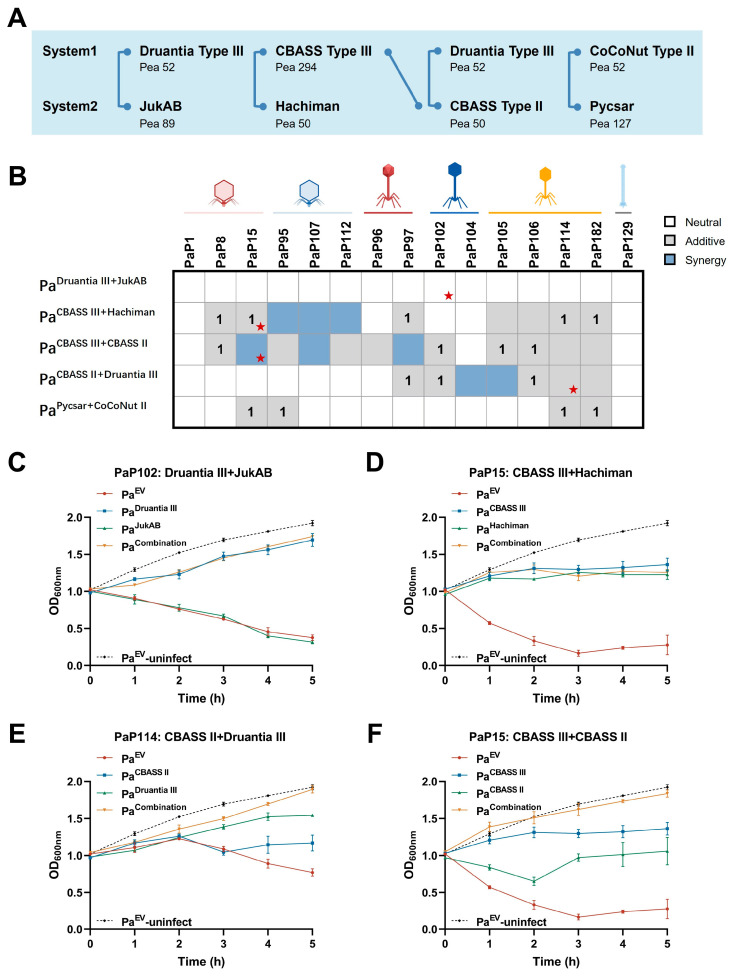
Combinations of defense systems provide a spectrum of interaction outcomes. (**A**) Schematic of the defense systems jointly cloned into *P. aeruginosa* strain PAO1. (**B**) Heatmap of combined effect provided by selected defense system pairs against phages compared to individual effects of the two parts. Neutral, |Log_10_ (EOP_combined_)| ≤ |Log_10_ (EOP_stronger_)|. Additive, |Log_10_ (EOP_combined_)| > |Log_10_ (EOP_stronger_)| but did not meet the threshold for synergy. Synergy, |Log_10_ (EOP_combined_)| > |Log_10_ (EOP_system1_)| + |Log_10_ (EOP_system2_)| + 1. This assay was independently repeated three times and only results consistent in at least two replicates were considered conclusive. The number “1” denotes only one order of magnitude defense enhancement over the stronger system (marginally additive, i.e., |Log_10_ (EOP_combined_)| = |Log_10_ (EOP_stronger_)| + 1). The asterisk denotes the group selected for the following analysis shown in (**C**–**F**). Bacterial growth curve under phage infection (MOI = 0.01) of (**C**) Druantia Type III, JukAB and their combinations with PaP102 infection, (**D**) CBASS Type III, Hachiman and their combinations with PaP15 infection, (**E**) CBASS Type II, Druantia Type III and their combinations with PaP114 infection, and (**F**) CBASS Type III, CBASS Type II and their combinations with PaP15 infection. Pa^EV^, PAO1 with empty vector pHERD20T. Pa^EV^-uninfect, the growth curve of PAO1 with empty vector pHERD20T without phage infection. All data are presented as the mean ± SEM of three biological replicates. Error bars in the figures denote the SEM.

Next, we sought to experimentally validate their functional interactions among defense systems identified in our co-occurrence analysis. Five pairs of significantly co-occurring defense systems were randomly selected from our collection of clinical *P. aeruginosa* strains (present in >15% of the clinical strains), including Druantia Type III-JukAB, CBASS Type III-Hachiman, CBASS Type II-CBASS Type III (r = 0.39, *p* = 3.88 × 10^−3^), CBASS Type II-Druantia Type III (r = 0.39, *p* = 3.88 × 10^−3^), and CoCoNut Type II-Pycsar (r = 0.33, *p* = 0.017) (Figure 4A, Table S4). Each system was cloned individually or in combination into the inducible expression vector pHERD20T and introduced into the model strain PAO1 (Figure 4A and Figure S3A). All the systems except JukAB conferred clear resistance against at least one-though not all-phages in the panel via different anti-phage mechanisms (Figure S3A–C). qRT-PCR verified successful induction of all systems in the plasmid-bearing PAO1 strains (Figure S4). 15 diverse lytic phages, which differed in their taxonomy (mentioned above), host ranges and adsorption ability, were employed to evaluate the resistance potency of defense systems (Figure 4B).

More than half of all 75 tested combinations (15 phages × 5 system pairs) showed neutral interactions (42 of 75, 56%), wherein the resistance of the co-expressed pair was statistically indistinguishable from the stronger single system. As expected, the neutral effect was observed in the pairwise combination involving JukAB. This is very likely attributed to the lack of a phage in our panel capable of activating JukAB, suggesting that functional interaction depends not only on the physical coexistence of both systems but also on their concurrent activation by a common threat. Despite the prevalence of neutrality observed across the phage-defense system interaction profile, four of the five tested pairs exhibited synergistic or additive effects against at least one phage. Synergy was not confined to phylogenetically distinct systems, as different subtypes of the same system CBASS family, Type II and Type III, displayed strong cooperative synergistic interaction when co-expressed.

To further validate the findings from our EOP assays, we assessed the impact of the defense system pairs on bacterial growth when infected with phages at an MOI of 0.01 in liquid cultures. We randomly selected four representative combinations: a neutral pair (Druantia Type III-JukAB pair with PaP102 infection), a marginally additive pair (CBASS Type III-Hachiman pair with PaP15 infection), a strongly additive pair (CBASS Type II and Druantia Type III with PaP114 infection), and a synergistic pair (CBASS Type III and CBASS Type II with PaP15 infection) (Figure 4C–F). The neutral pair and marginally additive pair showed a lysis delay profile largely similar to the stronger single system. In contrast, the strongly additive and the synergistic pairs conferred definitive survival advantages. In those two cases, co-expression led to significantly prolonged suppression of phage-induced bacterial lysis and thus sustained higher final cell densities compared to any single system. Altogether, this dynamic profile clearly explained that combinations of different systems did not necessarily confer a decisive survival advantage.

## 4. Discussion

In this study, we set out to elucidate the functional relationships between the diverse anti-phage defense systems in clinical *P. aeruginosa* and to determine how these interactions shape the overall phage resistance phenotype. Our genomic survey of over a thousand *P. aeruginosa* genomes reaffirmed its status as a species with an exceptionally rich and diverse arsenal of defense mechanisms [18,31,32]. The clinical isolates we sequenced further exhibited an enrichment in both the number and diversity of these systems compared to the broader RefSeq dataset, underscoring the intense selective pressures present in clinical environments. A pivotal and counterintuitive finding of our work was the lack of a significant correlation between the sheer number of defense systems in a strain and its innate level of phage resistance. This observation immediately suggested that the defensive outcome is not a simple sum of individual parts but is governed by more complex, qualitative interactions between co-resident systems.

Our co-occurrence analysis provided a systematic map of these potential interactions, revealing a network of statistically significant positive and negative associations among specific defense systems. This finding is conceptually aligned with recent work in other bacterial species [12]. Non-random co-occurrence and negative associations are widespread in nature, and crucially, some works provided experimental evidence for synergistic interactions between co-occurring pairs, including Gabija-tmn and Druantia III-Zorya II in *E.coli* [12], BREX and BrxU in *E. fergusonii* [26], and RM and CRISPR-Cas in *Staphylococcus aureus* (*S. aureus*) [28]. Our study in *P. aeruginosa* independently validates and extends this paradigm, demonstrating that such non-random genetic associations are a general feature of bacterial immune repertoires across distinct species.

However, our work introduces a critical nuance to this emerging model, as a more complex spectrum of outcomes were found in *P. aeruginosa*. The most prevalent interaction type we observed was neutral, where the combined effect of two systems was statistically indistinguishable from that of the stronger single system. This high frequency of non-additive interactions provides a crucial functional explanation for the genomic disconnect we observed: the acquisition of an additional defense system often confers no incremental fitness benefit against a given phage challenge. This prevalence of neutrality suggests that while synergistic pairs like CBASS II/III are powerful drivers of co-occurrence, many other co-inherited systems may not be functionally coupled or may require very specific, untested phage triggers for collaboration to manifest, as was potentially the case for JukAB in our panel [33,34].

The implications of our findings are manifold. First, from an evolutionary perspective, they underscore that the adaptive value of a defense system is not absolute but is contextual, depending heavily on the genetic background of the host. On the other hand, a defense system may be effective only against a narrow range of phages that utilize the very specific replication mechanism [6,11,35,36]. Therefore, the acquisition of a new system is not a guaranteed fitness improvement; its benefit is contingent upon its functional compatibility with the pre-existing defense network. This helps explain the remarkable heterogeneity in defense system repertoires even among closely related strains and suggests that “more” is not always “better” due to the prevalence of neutral interactions. Second, for the field of phage therapy, our results highlight a critical consideration. Predicting the susceptibility of a clinical pathogen based solely on the catalog of its defense genes is insufficient. The outcome of phage infection is dictated by the system-level interactions between these defenses. Understanding these interaction networks—identifying which pairs are synergistic, additive, or neutral—will be crucial for designing effective phage cocktails, for anticipating the evolution of resistance, and for devising strategies to circumvent these sophisticated bacterial immune networks. For example, phages could be selected or engineered to exploit gaps in these networks or to avoid triggering synergistic pairs. Moreover, this knowledge enables a more sophisticated strategy: a targeted antibiotic could first be used to selectively pressure the population, forcing a reliance on a specific synergistic defense pair, thereby creating a dependency that can be precisely exploited by a follow-on phage designed to disrupt that now-critical interaction.

A limitation of our study, shared with other works in the field [12], is the inability to exhaustively test all co-occurring pairs, especially those involving large and complex systems that are challenging to clone and express. Furthermore, the functional outcome of an interaction is phage-dependent, and our phage panel, while diverse, undoubtedly did not activate all potential defense pathways. Additionally, the comparative analysis between clinical and environmental niches was constrained by the limited metadata in public databases. Future research should expand the interaction map to include more systems and a wider array of phages, to delve into the molecular mechanisms that underpin these neutral and synergistic relationships, and to incorporate comprehensive, well-annotated collections of environmental isolates to further dissect the specific selective pressures exerted by the hospital niche compared to natural environments. In addition, the functional interaction map we have generated provides the essential dataset required to power predictive AI models. Such tools could integrate genomic features—such as defense system types, their genomic context, and co-occurrence patterns—to forecast whether a given *P. aeruginosa* strain’s resilience is governed primarily by the sheer abundance of its defenses or by the presence of critical functional interactions, thereby guiding more precise ecological forecasts and therapeutic interventions.

## 5. Conclusions

In conclusion, we establish that the anti-phage defense potential of *P. aeruginosa* is a complex emergent property, determined not by the quantitative sum of its parts but by the qualitative interplay between them. The prevalence of neutral interactions serves as a crucial reminder that genomic co-occurrence does not automatically translate to functional collaboration. By integrating genomic analysis with functional validation, we provide a systems-level framework for understanding prokaryotic immunity, with significant ramifications for both evolutionary biology and the translational pursuit of phage-based therapeutics.

## Data Availability

The raw sequencing data for the clinical isolates generated in this study have been deposited in the China National GeneBank Database (CNSA) with accession number CNP0008262.

## Supplementary Materials

The following supporting information can be downloaded at: https://www.mdpi.com/article/10.3390/microorganisms13122783/s1, Figure S1. Abundance and diversity of defense systems in *P. aeruginosa* genomes. (A) Comparison of number of defense systems between RefSeq and clinical isolates. Significance was evaluated using Mann–Whitney U test, * *p* = 0.0269. (B) Number of defense systems per genome in the RefSeq strain collection. The pink column indicates the median 9. (C) Number of defense systems per genome in the clinical isolates. The pink column indicates the median 11. (B) and (C) are related to (A). (D) Phylogenetic analysis for depicting the relationships among the 53 clinical strains and PAO1 reference genome. Pink represents Clade I, blue represents Clade II, purple represents Clade III. PAO1 is highlighted in yellow. The numbers adjacent to the branches represent the evolutionary distances. The phylogenetic dendrogram was constructed using the neighbor-joining method and a Poisson model in MEGA7, and tested by 1000 bootstrap replicates. The tree was subsequently visualized and annotated in iTOL. (E) Comparison of number of defense systems in the 53 clinical strains among phylogenetic groups. Significance was determined by One-way ANOVA with Bonferroni’s multiple comparisons test, * *p* = 0.0114. Figure S2. Network and counts of co-occurrence and negative association between defense systems. Related to Figure 3. (A) Network of co-occurrence between defense systems after Bonferroni correction. Nodes represent individual systems. Edges thickness is proportional to the absolute value of the correlation coefficient. (B) Counts of co-occurrence and negative association between defense systems. Significant correlations, adjusted by the Benjamini–Hochberg or the Bonferroni correction, are indicated by the color scheme in the legend. Figure S3. Defense systems cloned in *P. aeruginosa* strain PAO1 provide defense against a diverse range of phages. (A) Schematic of the defense system gene cassette cloned into *P. aeruginosa* strain PAO1. (B) Heatmaps of the order of magnitude change in phage titer, where phage titer is quantified by comparing the number of spots (with plaques, or clearing if plaques were not visible) on the tested strain divided by the Pa^EV^ strain. This assay was independently repeated three times, and only results consistent in at least two replicates were considered conclusive. Related to Figure 4. (C) Examples of plaque assays with phage on a lawn of PAO1 carrying different defense systems. Related to Figure S3B. Figure S4. Relative expression of core components of defense systems in PAO1 under overexpressed and basal conditions. The relative expression of components was normalized by its mRNA levels in cells without induction. Each experiment was repeated at least three times. Data are shown as mean ± SD. Statistical significance was determined by two-way ANOVA with multiple comparisons. ** *p* < 0.01 and *** *p* < 0.001. Figure S5. Growth curve of *P. aeruginosa* PAO1 carrying individual defense systems and their combinations in LB broth. (A) Druantia Type III, JukAB and their combinations. (B) CBASS Type III, Hachiman and their combinations. (C) CBASS Type III, CBASS Type II and their combinations. (D) CBASS Type II, Druantia Type III and their combinations. (E) CoCoNut Type II, Pycsar and their combinations. Pa^EV^, PAO1 with empty vector pHERD20T. Data are means ± standard error of means (SEMs) of three parallel samples for each trial. Table S1. Comprehensive list of defense systems in *P. aeruginosa* RefSeq (n = 1102). Table S2. Features of clinical isolates of *P. aeruginosa* used in this work, including defense system presence. Table S3. Features of *P. aeruginosa* phages used in this work. Table S4. Co-occurrence analysis and negatively association between defense systems in clinical *P. aeruginosa*. Table S5. The bacterial strains and plasmids used in this study. Table S6. Primers used in this study.
