# Supplementary material for: More Is Not Always Better: Co-Occurrence Analysis of Anti-Phage Systems Reveals Prevalent Neutral Interactions in P. aeruginosa
Source: Microorganisms. 2025 Dec 7;13(12):2783. doi: 10.3390/microorganisms13122783 (PMC12735699; doi:10.3390/microorganisms13122783)
Supplement: Supplementary file 1 [file microorganisms-13-02783-s001.zip › Supplementary figure_Han Shuhong.pdf]

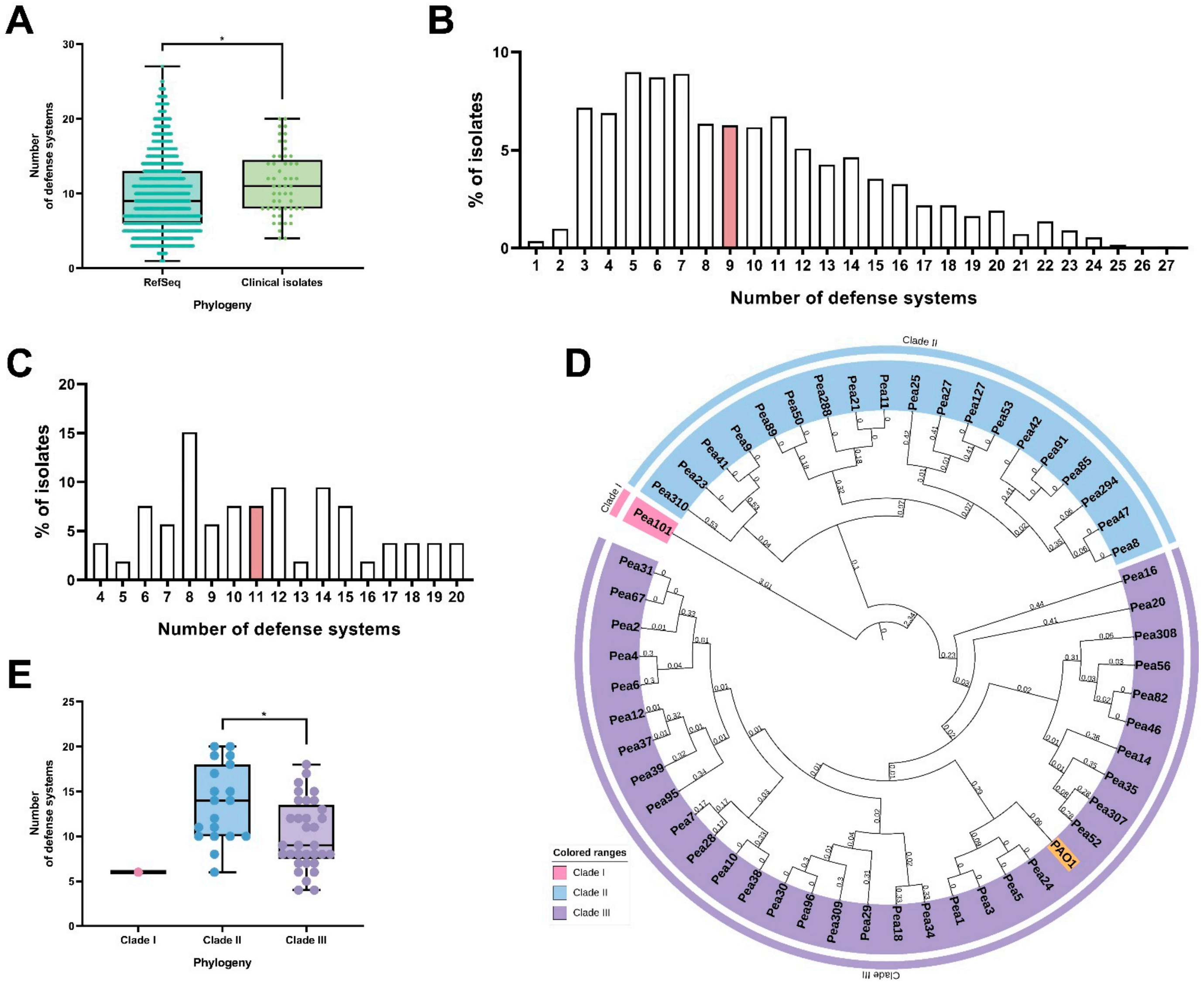

Figure S1. Abundance and diversity of defense systems in *P. aeruginosa* genomes. (A) Comparison of number of defense systems between RefSeq and clinical isolates. Significance was determined by Mann-Whitney U test, *p= 0.0269. (B) Number of defense systems per genome in the RefSeq strain collection. The pink column indicates the median 9. (C) Number of defense systems per genome in the clinical isolates. The pink column indicates the median 11. (B) and (C) are related to (A). (D) Phylogenetic analysis for depicting the relationships among the 53 clinical strains and PAO1 reference genome. Pink represents Clade I, blue represents Clade II, purple represents Clade III. PAO1 is highlighted in yellow. The numbers adjacent to the branches represent the evolutionary distances. The dendrogram was constructed using the MEGA7 and iTOL via the neighbour-joining method, with Poisson model, and tested by a bootstrap of 1000. (E) Comparison of number of defense systems in the 53 clinical strains among phylogenetic groups. Significance was determined by One-way ANOVA with Bonferroni's multiple comparisons test, *p= 0.0114.

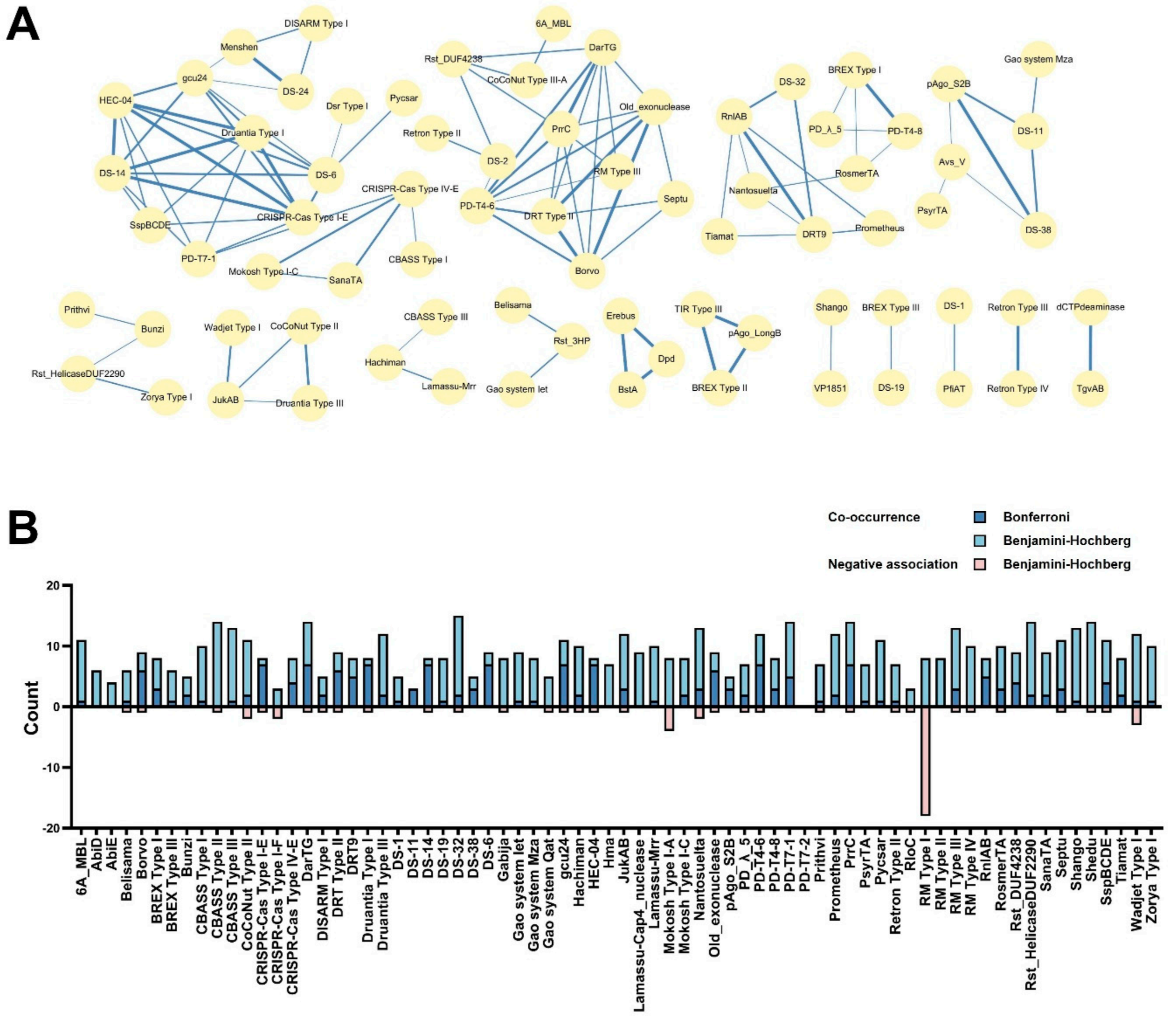

Figure S2. Network and counts of co-occurrence and negative association between defense systems. Related to Figure 3. (A) Network of co-occurrence between defense systems after Bonferroni correction. Nodes represent individual systems. Edges thickness is proportional to the absolute value of the correlation coefficient. (B) Counts of co-occurrence and negative association between defense systems. Significant correlations, adjusted by the Benjamini-Hochberg or the Bonferroni correction, are indicated by the color scheme in the legend.

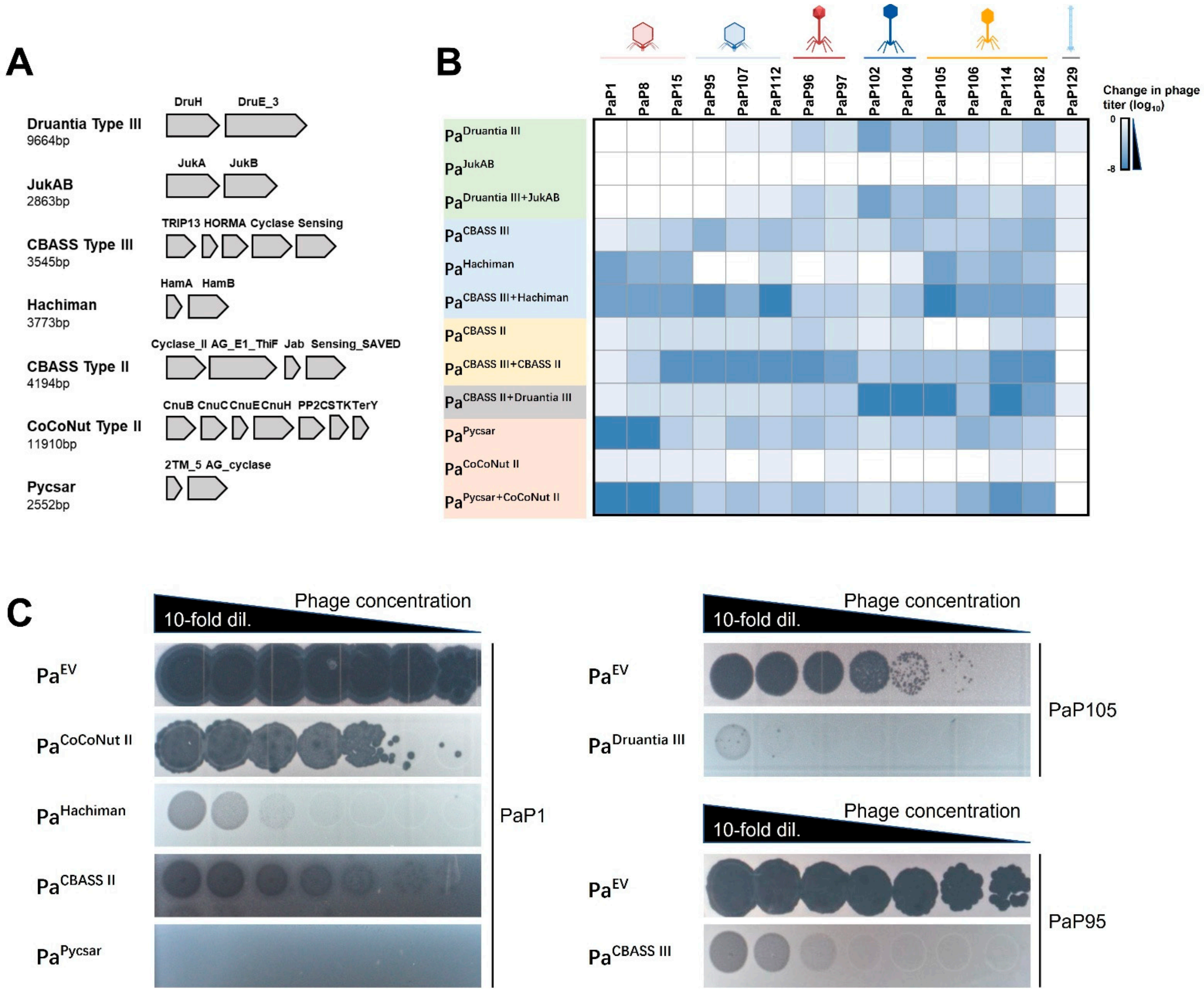

Figure S3. Defense systems cloned in *P. aeruginosa* strain PAO1 provide defense against a diverse range of phages. (A) Schematic of the defense system gene cassette cloned into *P. aeruginosa* strain PAO1. (B) Heatmaps of the order of magnitude change in phage titer, where phage titer is quantified by comparing the number of spots (with plaques, or clearing if plaques were not visible) on the tested strain divided by the Pa^{EV} strain. This assay was independently repeated three times, and only results consistent in at least two replicates were considered conclusive. Related to Figure 4. (C) Examples of plaque assays with phage on a lawn of PAO1 carrying different defense systems. Related to Figure S3B.

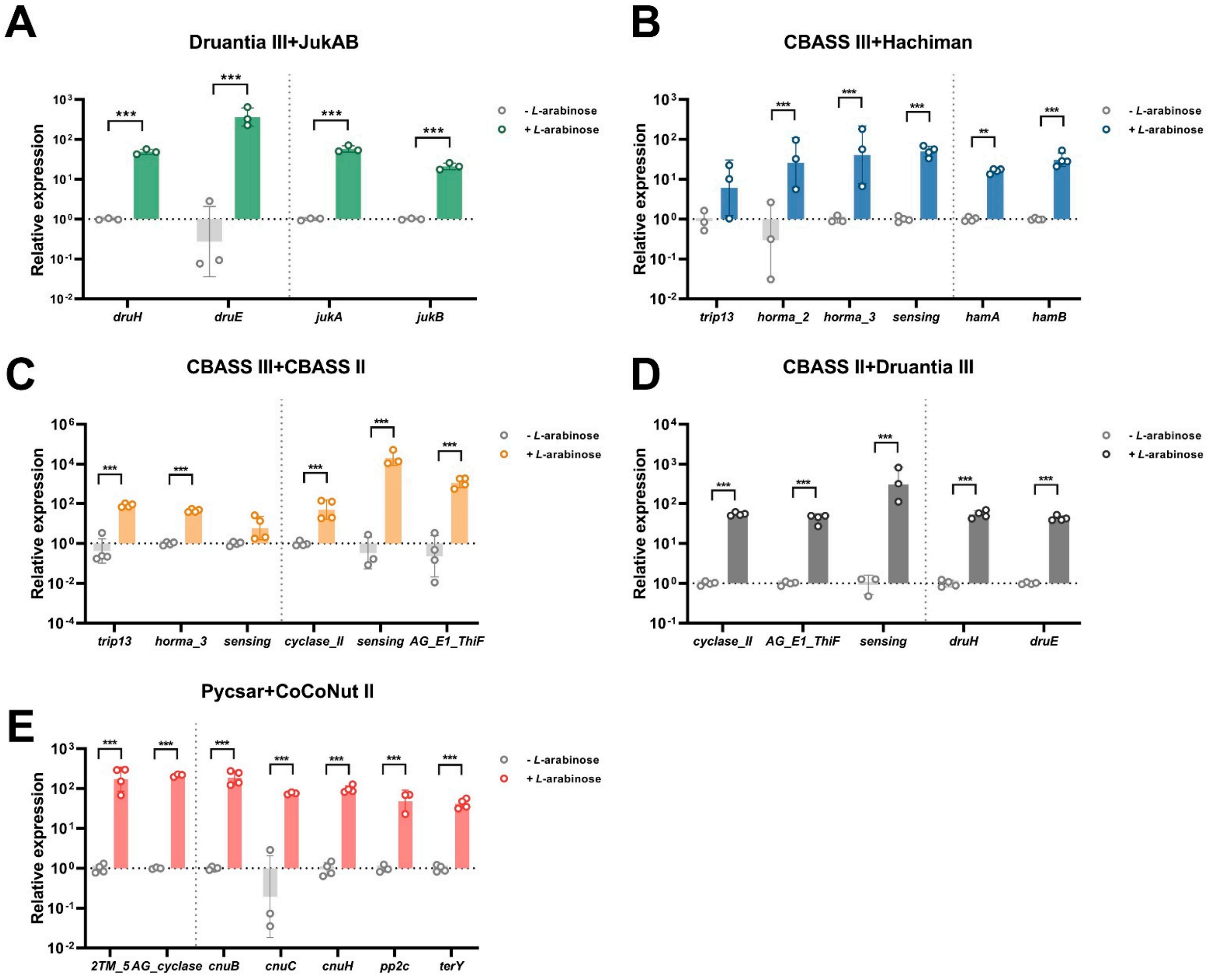

Figure S4. Relative expression of core components of defense systems in PAO1 under overexpressed and basal conditions. The relative expression of components was normalized by its mRNA levels in cells without induction. Each experiment was repeated at least three times. Data are shown as mean ± SD. Statistical significance was determined by two-way ANOVA with multiple comparisons. ***p* < 0.01 and ****p* < 0.001.

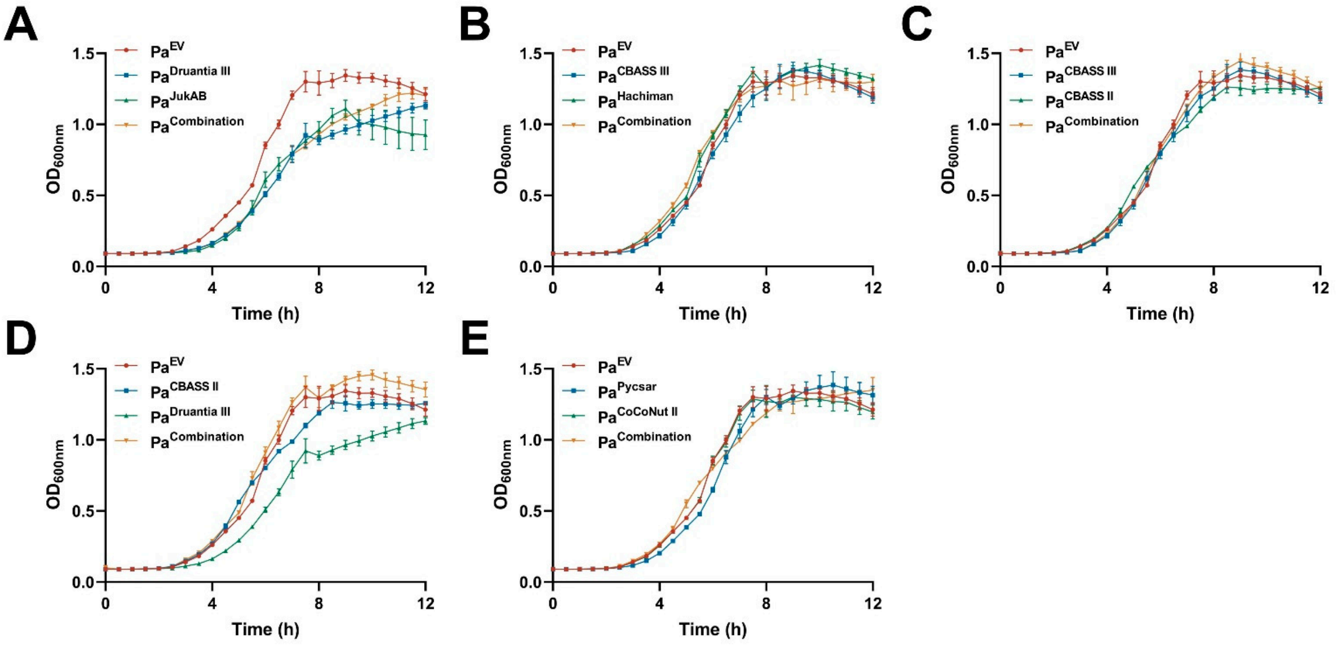

Figure S5. Growth curve of *P. aeruginosa* PAO1 carrying individual defense systems and their combinations in LB broth. (A) Druantia Type III and JukAB and their combinations. (B) CBASS Type III and Hachiman and their combinations. (C) CBASS Type III and CBASS Type II and their combinations. (D) CBASS Type II and Druantia Type III and their combinations. (E) CoCoNut Type II and Pycsar and their combinations. Pa^{EV}, PAO1 with empty vector pHERD20T. Data are means ± standard error of means (SEMs) of three parallel samples for each trial.
